# Principled Disagreements: Adhesion to Intergroup Justice Standards in the Context of the Belgian Linguistic Conflict

**DOI:** 10.5334/pb.345

**Published:** 2017-11-21

**Authors:** Olivier Klein, Pierre Bouchat, Assaad Azzi, Olivier Luminet

**Affiliations:** 1 Université Libre de Bruxelles, BE; 2 Université catholique de Louvain, Belgian Fund for Scientific Research, BE

**Keywords:** multi-ethnic conflict, intergroup relations, language, Belgium, distributive justice

## Abstract

According to the “Waffle” model of the Belgian Linguistic Conflict (Klein et al., 2012), this conflict centres around two main dimensions: One concerns the use of language across the territory and the second concerns the distribution of resources between the two main linguistic communities, Dutch-speakers and French-speakers. The model suggests that the two groups adhere to different justice principles regarding these issues and that these disagreements are a function of the intensity of the conflict. With respect to the first dimension, Dutch-speakers are expected to adhere more to a principle of linguistic territoriality than French-speakers who should be more in favor of a free choice of one’s idiom across the territory. With respect to the second dimension, the model posits that Dutch-speakers will adhere more to an equity principle whereas French-speakers should adhere more to a need principle. We tested these hypotheses in the context of a large-scale survey involving two waves: in May 2011 in the middle of a political crisis, and in June 2014, when the conflict was appeased. The pattern of “disagreements” in a subsample that participated in both waves of the survey (*N* = 378) is consistent with the Waffle model and, as expected, more severe at the heart of the conflict (in 2011) than after pacification (in 2014). However, differences were driven mostly by supporters of the Flemish nationalist party N-VA. Moreover, endorsement of principles on both dimensions are predictive of separatist attitudes in the Dutch-speaking sample whereas only the first dimension plays a role for the French speaking sample.

Many, if not most, modern states involve groups that differ in their ethnicity, language and/or religion. This raises the problem of how the state should allocate resources between such subgroups (Horowitz, 1985). When conflicts over resource distribution arise, the functioning of the state may be seriously altered. To take a contemporary example, in the aftermath of the terrorist attacks on Brussels on March 22, 2016, political scientist Dave Sinardet argued that the prolonged conflict over a territorial resource, the scission of the “Brussels-Halle-Vilvoorde” (BHV) electoral district along linguistic lines in Belgium (between 2009 and 2011 especially), prevented the Belgian State from adequately preparing for that foreseeable threat (Detaille, 2016). Other consequences of intractable conflicts can of course be violence or splitting of a previously unified state (Elcheroth & Spini, 2011). How are such conflicts appraised by citizens of such states? And how do such appraisals influence their aspirations for the future of the state? In this paper, we sought to address these conflicts through the lens of the Belgian linguistic context.

Given that this paper considers conflict over the distribution of resources between subgroups within a state, a definition is in order. A first useful classification distinguishes *material* from *nonmaterial* resources (cf. Azzi, 1998). A religious group may for example value the amount of funds it receives from the state to organize its activities (a material resource in countries, such as Belgium, that subsidizes religions). But it may also value the number of days off accorded to schools and workers for its holidays, a resource that is not directly tangible, such as funds or territory, but that acknowledges the group’s existence and importance. Acquisition of such a resource may be essentially geared at fulfilling symbolic needs.

The second distinction contrasts the distributive and the procedural component of resources allocation (Deutsch, 1985, Thibaut & Walker, 1975). Distributive justice considers the actual distribution of resources. But, groups are also preoccupied with the antecedents of these outcomes *i.e.*, how they were brought about. This *procedural* component concerns the process leading to these decisions. In the context of multi-group decision-making bodies, an important determinant of procedural justice judgment is the control exerted by one’s group over this process (Leventhal, 1980). For example in a legislative assembly, the number of members of one’s group who are represented in the body constitutes a crucial resource. We propose that an important part of procedural justice consists of the choice of the distribution principles to be used, a choice made through an agreement among representatives of the groups in the decision-making process.

That groups agree not only on the distribution of resources but also on the justice principles that are adopted to achieve this distribution, is at the heart of conflict escalation and de-escalation. This is in part because, in modern multi-ethnic (or multilinguistic) states, resources are distributed at the group level. Some of these resources, such as the number of religious holidays or language use, cannot be further subdivided between individual members of these groups. Others, such as tax income, can. How much each group receives, and on what basis, can naturally become a matter of contention.

What are the principles governing the distribution of resources in such societies? In intergroup relations, several principles have been considered (for a more comprehensive list, see Azzi, 1998). One, *equity* (Walster, Berscheid, & Walster, 1973), suggests that outcomes should be distributed between recipients as a function of each of these recipients’ contribution to the outcome. Another, *equality*, suggests that groups should be apportioned the same amount of resources regardless of their contributions. A third one, *need*, posits that groups should receive resources as a function of their needs.

In the present paper, we consider the predictors of adhesion to such principles in conflicts between groups sharing resources at a superordinate level. In such contexts, some groups at the subordinate level may differ in their contributions to the superordinate group, usually the State. These resources may then be redistributed at an individual or at a group level. For example, a tax rebate accorded to a taxpayer can be considered as an individual level contribution. Funds to create an airport on the other hand are likely to benefit a whole region, which may be sensitive when different ethnic groups are concentrated in specific regions.

How should such distributions be made? Two of the above principles merit special attention in this respect: equity and need (Deutsch, 1975). According to the first principle, people or groups who contribute more should earn more, receive more power or benefit from more services from the state. According to the second, people or groups who need it most, e.g., because they are more economically vulnerable, should receive more resources from the state.^1^

At a group level, equity can easily translate into demands for greater political or financial autonomy. Remember that this principle stipulates that individuals or groups who contribute more should receive more. Rather than having to provide resources to a less privileged outgroup, the privileged may aspire to an increased capacity to manage their own resources i.e., to exert greater control over the decisions that affect their outcomes. Such dynamics have been observed in the context of several regional conflicts such as Northern vs. Southern Italy, Spain (e.g., Catalonia, Basque Country), or Scotland (for a review, see: Friend, 2012). By contrast, less privileged subgroups may be more attracted to the need principle and to the preservation of the state allowing distribution of resources on this basis. Thus, endorsement of equity and aspiration to greater autonomy should be positively related whereas the opposite should be true for endorsement of need.

In the present paper, we consider how these principles unfold in the context of the Belgian linguistic conflict. After briefly sketching the country’s political situation, we consider in which ways appraisal of this conflict could inform more precise hypotheses regarding the interplay between multi-ethnic conflict and endorsement of justice principles.

## The Belgian Context

Independent since 1830, Belgium is a constitutional monarchy. It comprises two main linguistic groups: Dutch-speakers (+/– 60% of the population) and French-speakers (+/– 40%), with an additional small German speaking minority. The country is further subdivided administratively in three regions: Flanders in the North, which is officially Dutch-speaking, Wallonia in the South, which is mostly French-speaking and Brussels, which is officially bilingual, but predominantly French-speaking. Created as a centralized, unitary, state, Belgium has witnessed a series of reforms that have progressively transformed it into a federal state with large regional autonomy. The political evolution of Belgium has been marked by aspirations for greater autonomy from Flemish political movements and political parties, which their French-speaking counterparts have usually resisted before eventually making partial concessions. It is during these periods that the linguistic crises have been most acute. The latest such crisis has taken place after the federal legislative elections of June 2010, when 541 days were necessary to form a government (in December 2011) involving Dutch- and French-speaking parties (political parties are different on both sides of the “linguistic border”). The 6th reform of the state that ensued (voted in Parliament on December 19, 2013) granted more power to the regional entities at the expense of the federal government. Note that, contrary to other regional conflicts, the Belgian linguistic conflict has not witnessed significant episodes of political violence (for more information, see Dumont, 2012, Luminet et al., 2012).

## The “Waffle Model”: a Social Psychological Account of the Belgian Linguistic Conflict

In a previous paper (Klein, Licata, Van der Linden, & Luminet, 2012), we have put forward a social psychological model of the Belgian linguistic conflict, dubbed the “Waffle model”. This model suggests that this conflict involves two orthogonal dimensions.

The first dimension reflects the classical opposition between equity and need that we have already considered. This dimension concerns the distribution of economic resources between the linguistic groups. While bilingual Brussels and French-speaking Wallonia controlled the bulk of the economy until the 1950s, Flanders has progressively taken the upper hand and now contributes the largest share to the country’s finances: For example, in 2012, the per capita GNP was 38% higher in Flanders than in Wallonia (IWEPS, 2016). By contrast, the unemployment rate is much higher in Wallonia (12.5%) and Brussels (17.8%) than in Flanders (5.5%^2^). This raises questions as to how tax revenues (collected at the state level) should be redistributed. With respect to this dimension of the conflict, we suggested that equity and need played a significant role. Dutch-speakers are likely to adhere to the equity principle more than French-speakers: To the extent that Dutch-speakers contribute a greater share of the overall wealth of the country, the resources they receive from it should be proportional to this share. In the Belgian context, we (Klein et al., 2012) noted that equity often manifests itself through the belief that “work should be rewarded”. This is associated with stereotypes of Dutch-speakers as “hard working” and of French speakers as “lazy”. Conversely, French-speakers have argued that the resources of the state should be primarily targeted to the individuals and groups who most need it *i.e.*, thereby manifesting solidarity (an application of the need principle). Based on archival evidence, Klein et al. (2012) also considered the possibility that Walloons would be more likely to adhere to the principle of “temporal reciprocity” which stipulates that those who had benefited from contributions in the past should help their former contributors now that they are in need. This subsumes the presence of a belief that Wallonia has contributed to the development of Flanders (cf. Quévit, 1982). Note that in all cases, we suggest that group members adhere to the principles that maximize their interests, an observation that is common in the justice literature (cf. Thibaut & Walker, 1975), and in the Belgian context more specifically (e.g., Klein & Azzi, 2001).

The second dimension relates to the use of language across the territory. Between its independence in 1830 and the post-war period, Belgium has been ruled by a French-speaking bourgeoisie coming mainly from Brussels and Flanders (for a historical overview, see De Wever & Kesteloot, 2012). Yet, most of the working class spoke Flemish dialects and Dutch (in the North) or Walloon dialects and French (in the South). This has especially been an issue in Flanders, where the Flemish movement has resisted the use of French and highlighted its language as a central feature of its identity and a tool for emancipation. In this context, the Flemish movement has aspired to make Flanders an officially unilingual Dutch-speaking territory. By contrast, the French-speaking minority in Flanders has claimed a right to speak French, one of the three official languages (with Dutch and German), in its dealing with authorities especially in the municipalities situated on the Flemish territory where the French speaking population is dominant. Thus, this conflict highlights two opposing justice principles regarding the allocation of a symbolic resource: the use of language in interaction with local authorities over the territory. According to one view (*that we shall call territoriality*), only one language should be used by public institutions and authorities, namely the language of the region (i.e., Dutch for Flanders, French for Wallonia; Brussels being bilingual and the centre of recurring conflict). Hence, those for whom this is not the native idiom should adapt to the linguistic customs of their host region. According to the opposing view, that we shall call “*person based linguistic rights*”, all Belgians should be allowed to speak the language of their choice with public authorities all over the territory. Based on political discourses and historical evidence, we suggested that the French-speakers were more likely to adhere to this principle compared to Dutch-speakers, who were more likely to adhere to the rather assimilationist territorial principle: This responds in part to the fear of an expansion of French across the “linguistic border”, that was fixated in 1963 precisely to contain this “oil stain” (Rillaerts, 2010). French being the language of the upper classes all over the territory, French-speakers have resisted the learning of Dutch whereas Dutch-speakers had to learn French to achieve upward social mobility. The use of French within the Flemish territory could then be resented as a manifestation of disrespect for their collective identity (cf. Bourhis, Giles, Leyens, & Tajfel, 1979).

Note that these two dimensions concern two different types of resources: The first one (distribution of income and political power over this income) involves material (financial) and procedural (control) resources whereas the second one refers to a symbolic resource (use of language in official communication in the territory). If our reasoning is correct, Belgium is an ideal context for appraising the role of these two types of resources in multi-ethnic conflict. We propose three strategies for appraising the interplay between adhesion to such principles and intergroup conflict.

First, we are interested in differences between the two groups’ endorsement of these principles. As predicted by the “Waffle model”, Dutch-speakers are expected to endorse territoriality and equity more than French-speakers; and the latter are expected to endorse person-based linguistic rights, need and temporal reciprocity more than their counterpart.

Second, we expect endorsement of these principles to predict group members’ preferences for different outcomes to the conflict. In the Belgian case, several outcomes are conceivable. The most plausible ones are either a status quo, a greater autonomy of the regions or outright separation. Based on our analysis, endorsement of territoriality and equity should be associated with preferences for a less centralized state and hence for greater autonomy and separation.

Third, we expect intergroup differences in endorsement of these principles to be exacerbated at the height of the conflict compared to the more peaceful period that followed its resolution. According to the Waffle model, adhesion to these principles reflects group norms. Social identity theory (SIT: Tajfel & Turner, 1979) predicts that, when group identities are salient, such as in a time of intergroup conflict, people are more likely to define themselves as group members. They should therefore be more likely to “self-stereotype” themselves and endorse these norms. This is a central prediction of self-categorization theory (Turner, Hogg, Oakes, Reicher, & Wetherell, 1987), a later elaboration of SIT.

## The Present Study

In order to test the current hypotheses, we compare the endorsement of several justice principles in a sample composed of members of the two main Belgian linguistic groups during the Belgian political crisis of 2010–2011 and in 2014, when it was appeased after the formation of a government that was able to reach a new agreement on reforming the state. In the present study, we focus on a small subsample of participants who took part in the two waves of the study. This allows controlling for possible differences in sample composition from time 1 to time 2 and to more confidently make claims regarding causality. The principles we are considering are the following: Need, equity (in general and applied to work especially), Temporal reciprocity, Territoriality and Person-based linguistic rights.

## Method

### Sample

Studies were announced on the website of two universities, one for each linguistic group (Université libre de Bruxelles and Katholieke Universiteit Leuven). In addition, announcements were made on major newspapers websites (*De Standaard* and *Le Soir*) for the 2011 data collection. The participants were invited to complete an online questionnaire that was presented in a bilingual version (the order of appearance of the languages was counterbalanced across the questionnaire from one scale to the next). Data collections took part respectively between May 6th and June 2nd 2011 and between May 5th and May 24th 2014. The first period correspond to the longest political crisis in Belgium. Indeed, after the elections of June 13, 2010, which were won by the Flemish Nationalist party N-VA (on the Dutch-speaking side) and by the Socialist Party, PS (on the French speaking side), the heads of Belgium’s political parties did not succeed in forming a coalition. Most Flemish parties demanded greater autonomy for their Region through a reform of the State and the scission of the bilingual judicial district of BHV, which requires a large majority (2/3 of members of parliament). Note that these demands relate to both the first (economic) and to the second (linguistic) dimension of the Waffle model. French speaking parties were initially reluctant to grant these demands. An agreement on such a reform was however achieved in 2011 and a government formed in December 2012 (without the N-VA). Hence, the second period, during which this government was still in power, can be qualified of a post-crisis period, when the linguistic divide was much less salient. For 2011, N = 2806 (1228 or 40.2% French). For 2014, N = 1909 (1257 or 65.84% French). The reliability of the scales was computed on the overall sample. However, to test our hypotheses, we focus only on 378 participants who took part in the two waves and were contacted via email for the second wave (a rate of 22% based on the 1749 email addresses available for 2011).^3^ Only participants who self-described as French or Dutch speakers were included (i.e., not the “Bilingual” or “Other”). These were divided as follows: 265 (68%) female and 113 male, 223 (59%) Dutch-speakers and 155 French-speakers, *M_age_* = 43, *SD* = 16.

We asked participants to express to which party they felt closest in a list of the main Belgian parties. They could check multiple responses. The response frequencies are reported in Appendix 1. As is clear from this table, a sizable number of participants (especially on the French-speaking side) express sympathy for parties of the other linguistic group (although it is not possible to vote for them, except in Brussels). In terms of political orientation (1 = far left, 7 = far right), the groups did not differ reliably although the French-Speaking group was slightly more left-wing (*M_French_* = 3.80, *M_Dutch_* = 4.16, *t*(310)^4^ = 2.25, *p* = .025, *d* = .26) which corresponds to the votes at the national level. Support for the Flemish nationalist party N-VA was over-represented in the Flemish sample compared to the general population whereas support for the Socialist Party was underrepresented in the French-speaking sample.^5^ Overall, endorsement of the green parties was also higher than in the general population (based on Election results).^6^

### Measures

The measures we consider in this paper are part of a larger questionnaire. The full questionnaire is accessible at osf.io/34xta.

**General information.** Respondents indicated their age, sex, education and occupation, and the postal code of their residence in addition to their mother tongue and the political party affinity measure described above.

**Principles.** Participants were told the following “Find below several principles. Please indicate for each value or principle, to what extent it is important for you personally in the Belgian context”. Responses were made on 7-point scales (1 = “not important at all”, 7 = “very Important”). Below is the wording of each item and their labels:

Need was captured with two measures: “Solidarity between individuals” (labeled Solidarity) and “In a country, resources must go first to those who need them most” (Need). Equity was measured with “In a country, each person should receive resources as a function of his or her contribution to this country” (Equity) and “The less one works, the lesser money one should receive” (Work Equity). Temporal reciprocity was measured with “In a country, if one has received resources in the past, it is normal to give them back later”. The second, dimension was captured with two items: “Each person must adapt to the uses and customs of the territory in which s/he lives” tapped adhesion the territorial principle (Territoriality). Adhesion to person-based linguistic rights was assessed with the following item: “Everyone should be allowed to speak his or her language anywhere in the Belgian territory” (Person-based LR).

**Endorsement of separatism.** Participants were asked to estimate how desirable they viewed the following outcome “Belgium will be separated in two independent states” within the next five years (1 = “not desirable at all”, 7 = “highly desirable”).

## Results

### Adhesion to Justice Principles

We first computed the correlations between adhesion to the seven justice principles. These correlations are reported in Table 1 (for 2011 and 2014 separately). A glance at these correlations suggests that most are significant and stable over time. Note also that, in line with our model, the items tapping equity on the one hand and need on the other are negatively correlated, as are territoriality and person-based linguistic rights. By contrast, correlations within dimensions (equity and need) are in the expected direction. However, since the measures are clearly distinct (the maximum coefficient is .51), we decided to analyse them separately. Hence, when we refer to “need” and “equity” in this section, we allude to the individual items described above (as we do for “solidarity” and “work equity”).

**Table 1:** Spearman Correlations Between Adhesion to Different Principles and adhesion to separatism.

	Solidarity	Need	Equity	Work Equity	Temporal reciprocity	Territoriality	Person-based LR	Separatism

Solidarity	.61**	.48**	–.22**	–.25**	–.10	–.20**	.33**	–.38**
Need	.54**	.59**	–.30**	–.30**	–.14	–.18*	.17**	–.19**
Equity	–.31**	–.35**	.56**	.51**	.49**	.39**	–.10	.15*
Work Equity	–.24**	–.28**	.35**	.55**	.35**	.39**	–.07	.15*
Temporal reciprocity	–.22**	–.21**	.47**	.28*	.50**	.28**	.00	.20**
Territoriality	–.27**	–.26**	.35**	.35**	.22**	.58**	–.21**	.25**
Person-based LR	.39**	.20**	–.16**	–.06**	–.04	–.34**	.64**	–.26**
Separatism	–.42**	–.21**	.28**	.20**	.21**	.33**	–.37**	.75**

*Note*: Below the diagonal: 2011, Above the diagonal: 2014. Diagonal: Between 2011 and 2014. *: *p* < .05 **: *p* < .01

**Figure 1:**
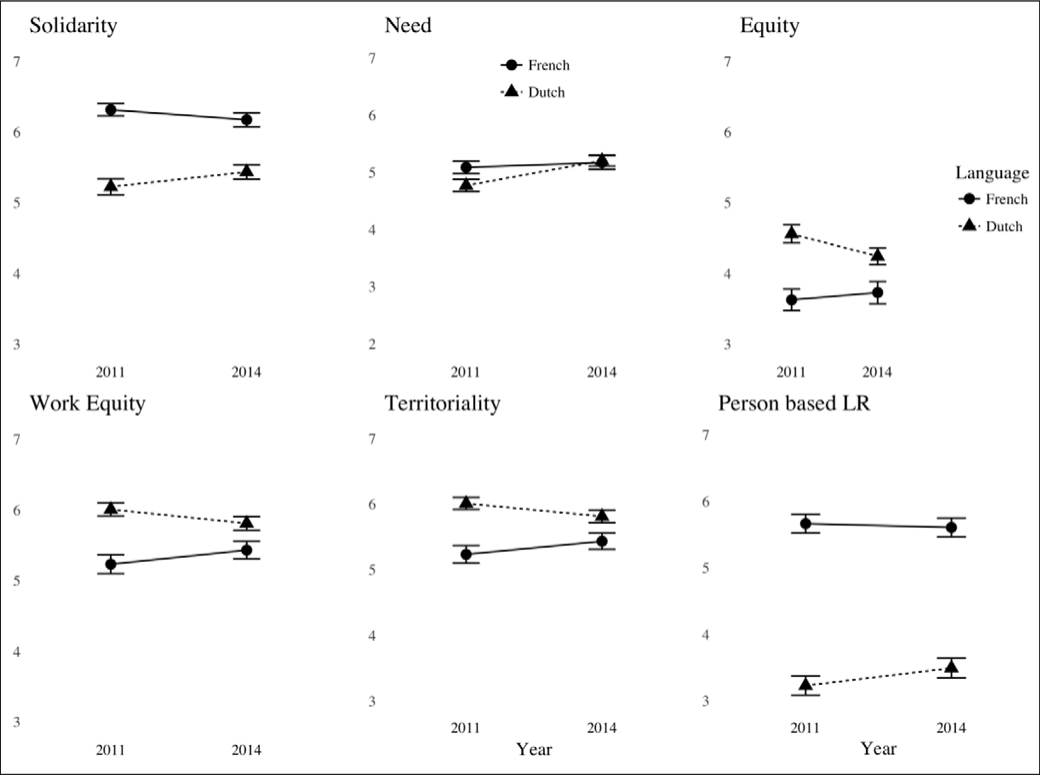
Mean endorsement of six principles as a function of Year and Linguistic Group.

To examine the impact of linguistic group and time (2011 vs. 2014) on endorsement of these principles, we conducted mixed analyses of variance on each item with time as a repeated measure and language as a between-subject variable. In all cases, we introduced age and gender as covariates. We then computed simple effects of time separately for the two language groups. Results of these analyses are reported in Table 2. The corresponding means are reproduced in Figure 1 for all dependent variables except Temporal Reciprocity, for which no effect was observed (*M* = 3.77, *SD* = 1.82).

**Table 2:** Results of Mixed Analyses of Variances.

Effect	Statistic	Solidarity	Need	Equity	Work Equity	Temporal reciprocity	Territoriality	Person-based LR

Age	*F*(1,374)	1.16	.33	6.15*	4.50*	7.97**	13.07**	.98
Gender	*F*(1,374)	20.82**	1.11	.40	3.31^+^	2.42	2.15	13.41**
Language	*F*(1,374)	42.73**	.79	18.39**	7.47**	1.55	18.38**	146.65**
Year	*F*(1,376)	.93	17.9**	2.48	4.80	.18	.24	1.72
Language × Year	*F*(1,376)	6.40*	6.30*	5.03*	1.55	.38	7.64**	2.47
Simple effects of Year								
Dutch speakers	*F*(1,222)	4.46*	22.10**	7.33**	5.84*	.01	6.23*	3.63^+^
French Speakers	*F*(1,154)	2.75^+^	.68	.48	.22	.47	2.38	.16

*Note*: ^+^: *p* <.10, *: *p* < .05, **: *p* < .01.

Analyses of main effects reveal that Dutch-speakers tend to adhere more to two principles than French-speakers i.e., equity (in its two forms) and territoriality. The reverse holds for solidarity, and person-based LR, for which a massive effect is found (see Figure 1). These trends are consistent with our hypotheses. By contrast, no effect of language is found for need.

However, there was an interaction between linguistic group and time on need with Dutch speakers becoming more favorable to this principle over time (see Table 2). The same holds for the closely associated principle of solidarity. Interactions on equity, work equity and territoriality revealed the reverse trend: Dutch-speakers became less favorable to these principles. With respect to the latter variable, a simple main effect of time was also observed on the French-speaking group, who grew more favorable to this principle. Whereas no interaction was observed on person-based linguistic rights, it is worth noting that an effect of time was found when focusing on the Dutch-speaking group only: They were more favorable towards this principle in 2014 than in 2011. More generally, an inspection of Figure 1 reveals that Dutch-speakers grew more sympathetic to principles that were more popular in the French group and less sympathetic towards principles advocated by their group (according to the Waffle model). Although simple effects were generally not significant for French-speakers, we see that, from a descriptive point a view, they became less favorable to solidarity and need and more favorable towards equity and territoriality, moving closer to the “Dutch-speaking” perspective.

We made the same analyses on the part of the sample that was involved in one wave only. Indeed, given that this is the majority of the sample, there is a legitimate concern that the previous results may be unrepresentative given the high dropout rate. The effects evidenced in the smaller sample were all reproduced in the larger sample although a few additional effects emerged due to greater power. Of greatest interest, we found a very small, but significant, interaction between linguistic group and time on the temporal reciprocity variable, *F*(1,3708) = 25.37, *p* < .01, *η_p_^2^* = .006. Thus, the 2014 Dutch-speaking sample was less favorable to this principle than the 2011 one (*M_2011_* = 4.16, *SD* = 1.74, *M_2014_* = 3.74, *SD* = 1.66) whereas the reverse happened for French speakers (*M_2011_* = 3.85, *SD* = 1.87, *M_2014_* = 4.21, *SD* = 1.82). Another notable finding was observed on the need variable, where the interaction between linguistic group and time (*F*(1,3707) = 11.27, *p* < .01, *η_p_^2^* = .002) resulted in a reversal of the linguistic difference. Whereas, in 2011, French speakers were more favorable to this principle than Dutch speakers (*M_French_* = 4.94, *SD* = 1.87, *M_Dutch_* = 4.77, *SD* = 1.75), this difference reversed in 2014 (*M_French_* = 4.94, *SD* = 1.82, *M_Dutch_* = 5.20, *SD* = 1.66).

The interaction between linguistic group and time on the linguistic rights principle also reached significance in the larger sample, *F*(1,3708) = 10.98, *p* < .001, *η_p_^2^* = .002). The difference between French and Dutch-speakers decreased between 2011 (*M_French_* = 5.60, *SD* = 1.87, *M_Dutch_* = 3.33, *SD* = 1.74) and 2014 (*M_French_* = 5.64, *SD* = 1.82, *M_Dutch_* = 3.97, *SD* = 1.66) due to the latter’s greater endorsement of this principle (simple effect of time: *F_Dutch_*(1,1536) = 14.60, *η_p_^2^* = .01; *F_French_* < 1).

### Controlling for Political Preferences

Given that our subsample is not representative, it is conceivable that some of these differences are driven by imbalances in political preferences between the two groups. To appraise this possibility, we coded participants’ political affiliation in six categories: Green (Groen & Ecolo, *n* = 71), Christian Democrat (CD&V & CDH, *n* = 48), Socialist (SPa & PS, *n* = 31), Liberal (open VLD & MR, *n* = 61), Nationalist (N-VA, *n* = 121) & Other (*n* = 46). The first six categories included people who had chosen either one or two parties in each “pair”. The “Nationalist” category included participants in the rest of the sample who had chosen “N-VA” (either in isolation, *n* = 84, or in combination with 1 or 2 other parties, *n* = 26). The “other” category grouped people who had not chosen any party (*n* = 9), chosen FDF (n = 9, of which 7 in isolation), Vlaams Belang (n = 2), named another unlisted party, such a PTB-PdvA (*N* = 23, of which 2 combined with another).^7^ The rest included people who had chosen two or more parties that did not all belong to one of the above “families”, e.g., such as SP.a and open VLD (*n* = 17). We then ran a series of linear mixed model using the lme4 package in R (Bates, Mächler, Bolker, & Walker, 2014). This model included the same effects as the previous ANOVAs but in addition to random effect of participants’ id, it included political affiliation as an additional fixed effect. This amounts to performing an analysis of covariance controlling for party preference (cf. Snijders & Bosker, 2012). To introduce this variable in the model, it had to be contrast coded by introducing five dummy variables (see Table 3). Gender (Male = –1, Female = 1), Year (2011 = –1, 2014 = 1) and Group Membership (French-speaking = –1, Dutch-speaking = 1) were contrast coded as well. The interaction between year and group membership was also introduced in the model. The statistical significance of these effects was tested using the Satterthwaite approximation (Satterthwaite, 1946) via the LMERtest package in R (Kuznetsova, Borckhoff, & Christensen, 2015). Below, we report the main results of those analyses, and especially those that qualify the preceding analyses. The full results are available here: https://osf.io/ymu6p/.

**Table 3:** Dummy Variables Used to Code Political Affiliation.

Contrasts	Green	Socialist	Liberal	Christian Democrat	Nationalist	Others

1. Nationalists vs. Others	–1	–1	–1	–1	5	–1
2. Green vs. Rest	4	–1	–1	–1	0	–1
3. Others vs. Liberal/Socialist/Christian Democrats	0	–1	–1	–1	0	3
4. Christian Democrats vs. Liberal/Socialists	0	–1	–1	2	0	0
5. Liberals vs. Socialists	0	–1	1	0	0	0

Unsurprisingly, for all principles, the political affiliation variable exerted a significant effect (*p* < .01). Second, the first contrast opposing Flemish nationalists to all other affiliations was significant as well. Nationalists endorsed equity (*B* = .18), work equity (*B* = .18) and territoriality (*B* = .23) more and endorsed solidarity (*B* = –.23), need (*B* = –.17), and person-based LR (*B* = –.21, all *p*s < .01) less than participants with other political preferences.

Next, we consider changes affecting the conclusion of the effects of our core predictors (language and year).

**Figure 2:**
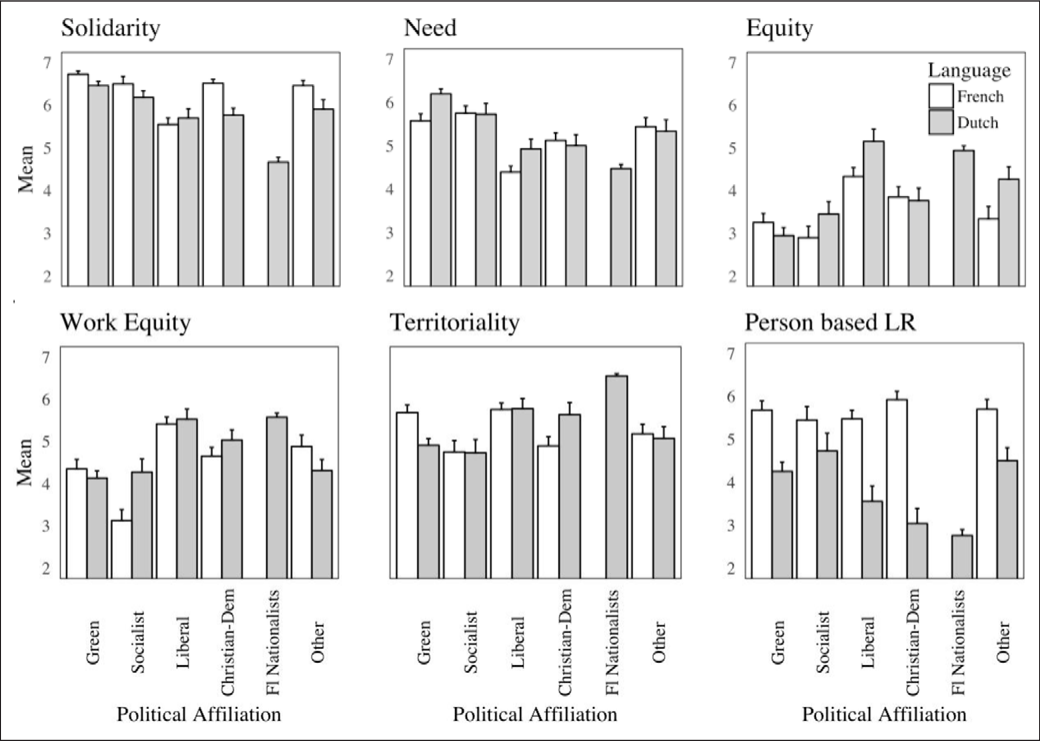
Endorsement of justice principles as a function of party affiliation and group membership.

First, the main effect of language on the solidarity variable became marginally significant, *B* = –.13, *p* = .051. When considering each political affiliation separately (see Figure 2), we note that the simple effect of linguistic group is significant for Christian democrats only if we use separate mixed ANOVAs within each political affiliation, *F*(1,46) = 12.6, *p* < .01, *η*^2^ = .21). It is also marginally significant in the “Other” category (*F*(1,44) = 3.14, *p* = 0.083, *η*^2^ = .07).

Second, on the need principle, we witness a very interesting *reversal* of the Language effect. Thus, controlling for political affiliation, Dutch-speakers are actually *more* favorable to this principle than French-speakers, *B* = .14, *p* = .053. An inspection of Figure 2 reveals that this is because Flemish nationalists, who are very numerous in the sample, and almost exclusively Dutch-speaking, tend to be opposed to this principle. Among greens and liberals at least, support for the need principle is higher in the Dutch-speaking than in the French-speaking sample (*F*(1,69) = 7.05, *p* < .01, *η*^2^ = .09 for Greens and *F*(1,59) = 3.22, *p* = .078, *η*^2^ = .05 for liberals, using separate mixed ANOVAs within each political orientation).

Third, the effect of group membership observed on the equity and work equity principles became non significant (*Bs* = .18, *p* = .14 and *B* = .001, *p* = .73 respectively). To appraise this change, we inspected the mean endorsement of these principles as a function of linguistic group and political affiliation (see Figure 2). With respect to equity, we note that for socialists, liberals, and “others”, the difference is in the expected direction (although it is marginally significant for the latter two groups only using mixed ANOVAs, *F_Liberals_*(1,59) = 3.63, *p* = .06, *η*^2^ = .06, and *F_Others_*(1,44) = 2.89, *p* = .096, *η*^2^ = .06). Among greens, the difference is in the opposite direction (although not significantly so) and among Christian democrats, no difference is observed. With respect to work equity, we note that endorsement of this principle is significantly higher among Dutch-speakers for socialists only, *F*(1,29) = 5.52, *p* = .026, *η*^2^ = .16 (see Figure 2). Among people who prefer other political orientations, no significant difference is observed as a function of linguistic group.

Fourth, the main effect of linguistic group on the territorial principle became non significant (and even changed sign) as well, *B* = –.05, *p* = .63. To understand this change we examined the effect of linguistic group within each political group affiliation using separate mixed ANOVAs (including main effects of Language and Year). Although among socialists, christian democrats and liberals, Dutch-speakers consistently espoused this principle more than French speakers, none of these “simple effects” reached significance (alpha = .05). However, within the sizeable green group, French-speakers were *more* in favor of this principle, *F*(1,69) = 8.08, *p* < .01, *η*^2^ = .10. Again, the mean for the Flemish nationalists was much higher than the rest of the sample (see Figure 2). Taken together these opposite trends within two political orientations heavily represented in our sample explain that the linguistic effect was cancelled when taking this variable into account.

Finally, note that, unlike its influence on the main effects of linguistic group, introducing political affiliation in the model did not affect the interpretation of the interaction terms involving year and linguistic group. Interactions on solidarity, need and territoriality remained significant. The effect of linguistic group on personality based LR remained highly significant as well, *B* = –.84, *p* < .001.

### Prediction of Separatism

Endorsement of separatism at time 2 (2014) was very skewed (*M* = 2.67, skewness = .89, kurtosis = 2.3). In the Flemish sample, 48% of participants chose 1 (“not desirable at all” *vs.* 73% for French-speakers) and 16% 7 (“highly desirable” vs. 7% for French-speakers). As expected, a Wilcoxon’s rank-sum test showed that Dutch-speakers adhered more to separatism than French-speakers, *W* = 12.200, *p* < .001. In order to examine whether endorsement of justice principles predicted this variable, we relied on an ordered logistic regression, which does not make any assumptions about the distribution of the dependent variable. In each linguistic group, we examined whether endorsement of the justice principles at time 1 (2011) predicted the endorsement of separatism at time 2 (2014) using the *polr* command from the MASS package in *R* (Ripley, 2016). In each case, we fitted two models: In model 1, we only included the justice principles as predictors; in model 2, we also entered endorsement of separatism at time 1. The latter allows us to examine whether justice principles add explanatory power over and above “baseline” levels of separatism at time 1. Results of these analyses are reported in Table 4. It appears from this table that, in the Dutch-speaking group, solidarity, and person-based linguistic rights both strongly predict endorsement of separatism negatively whereas territoriality predicts it positively. However, when separatism at time 1 is controlled, only need and work equity emerged as a predictor of separatism at time 2: The more Dutch speakers endorse need, the less they endorse separatism. Note also an unexpected gender effect: Dutch speaking women seem to reject separatism more than their male counterparts.

**Table 4:** Ordered Logistic Regression: predicting attitudes towards separatism in 2014.

	Dutch speaking		French speaking	
	
	Model 1	Model 2	Model 1	Model 2

Predictors				
Separatism (2011)	–	2.07**	–	1.52**
Age	.31*	.29	.16	.30
Gender (Female)	–.46*	–.42*	–.26	–.27
Solidarity	–.37**	.25	–.64*	–.50
Need	–.13	–.49*	.13	.12
Equity	.14	–.28	–.24	–.23
Work Equity	–.07	–.38*	–.19	–.35
Temporal reciprocity	.21	.33^+^	.30	.37
Territoriality	.67**	.30	–.11	–.07
Person Based LR	–.33*	.08	.00	.20

AIC	645.38	522.45	327.48	290.04
Residual Deviance	615.38	493.45	297.48	258.04

*Note*: The standardized coefficients refer to the predictions of adhesion to separation in 2014 based on adhesion to each of the 7 principles in 2011. ^+^: *p* <.10, *: *p* < .05, **: *p* < .01 for tests that the coefficients is equal to 0 in the population.

In the French group, model 1 reveals that solidarity is related (negatively) to endorsement of separatism. This effect disappears when including separatism at time 1 as a predictor (cf. Table 4).

## Discussion

The purpose of this paper was to compare the endorsement of several justice principles governing the allocation of resources between citizens in Belgium. Specifically, based on the “Waffle model”, we predicted that the more affluent Dutch-speakers would adhere more to equity and less to need than the French-speakers. This prediction was confirmed. Contrary to expectations, adhesion to temporal reciprocity was not affected by language, at least in the sample that took part in the two waves. When considering the whole sample, French-speakers were more favorable to it in 2014 than in 2011 whereas the Dutch-speaking groups displayed the reversed pattern. This is contrary to our expectations. The lack of clear results on this item may be due to its wording, which does not specify a clear timeframe. Thus, whereas French-speakers may have the feeling that “they” helped Dutch-speakers in the early XX^th^ century, the latter may perceive that they have been assisting Wallonia and Brussels for many more years. On the second, territorial, dimension of the model, we find, as expected that Dutch-speakers adhere significantly more to territoriality and less to personality based linguistic rights than French-speakers. The latter effect especially is extremely strong.

We also obtained evidence that adhesion to these principles varies as a function of time. This is true at an individual level, where we find moderate correlations between adhesion of justice principles at time 1 and at time 2 (see Table 2). It is also true at the group level, but only in the Dutch-speaking group who, over time, becomes more likely to view that resources should be attributed to those who need it most, and less on the basis of the amount of work recipients have invested to acquire these resources (a form of equity). On the “territorial” axis, we notice that they are less likely to endorse a territorial principle according to which all citizens of one of the countries’ regions should adapt to its customs and more likely to tolerate the use of multiple languages in official communication in each region. Thus, the Dutch-speaking group seems to have moved in the direction of the traditional attitudes of the French-speakers. Although the French-speakers’ endorsement of justice principles seems more stable, they exhibit a greater endorsement of territoriality in 2014 than in 2011. The Waffle model predicted a greater convergence between the two groups as a consequence of the de-escalation of the conflict between 2011 and 2014. Our findings are generally consistent with this prediction.

These results suggest that attitudes towards justice principles depend on the intergroup context. Previous research (Rimé, Bouchat, Klein, & Licata, 2015) has already shown that group differences in the endorsement of separatism in Belgium differed across generations. The present findings confirm that the two groups’ political attitudes are amenable to change.

We also find evidence that endorsement of justice principles predicts adhesion to (or rather rejection of) separatism, which remains a minority choice in both subsamples (although it is more popular among Dutch-Speakers). Specifically, when controlling for separatism at time 1, we find that principles associated with the first dimension of the model predict separatism at time 2 (i.e., need and work equity) and we find this in the Dutch-speaking group only. This observation is consistent with the notion that, with the increased prosperity of Flanders and the achievement of a territorial separation, the “old” nationalism anchored in resentment towards the French speaking elite encroaching on Flemish soil has given way to an economic one focused on greater political and financial autonomy (Huysseune, 2011).

Finally, it is worth noting that while we tended to view solidarity and need as two manifestations of the same principle, the pattern of results on these two items is quite different. Thus, intergroup differences are much higher on the “solidarity” than on the “need” item. This leads us to reconsider the conceptual underpinnings of these concepts. The concept of solidarity, more than the concept of need, presupposes the existence of a common or implicit ingroup, defining to whose needs one must be responsive (Hogg & Hains, 1996). It is associated with a sense of group cohesiveness. Differences between these two variables may therefore be a function of a greater endorsement of Belgium as a meaningful in-group for the French-speakers. This would explain why solidarity negatively predicts separatism better than need does.

## Role of Political Preferences

We found that some of these effects of linguistic group on endorsement of justice principles were qualified by the introduction of political preferences as a “covariate”. This suggests that differences in party affiliations, rather than linguistic group per se, explain these differences. Especially, we found that the main effects of linguistic group observed on equity, work equity, solidarity and need were driven to a great extent by supporters of the N-VA. Actually, this group’s position tends to differ from all other groups even when taking language into account.

Given that party affiliations are driven in part by preferences for justice principles, this should not be construed as a “confound”. For example, while the number of N-VA supporters in our sample probably exceeds its proportion in the Belgian population in 2011, effects driven by this group reflect the very opinion dynamics in the Dutch-speaking population that our model seeks to explain.

On the territorial principle, which prescribes the adoption of the language and customs of the territory in which one lives, we note an interesting pattern when taking political preference into account: Only in the Christian democrat “family” do we find an effect of language. And not surprisingly, the Flemish nationalists are the greatest supporters of this principle. Note that these two political movements have been the greatest drivers of Flemish autonomy (cf. Delwit, 2012). However, unexpectedly, we find that among Green parties, the territorial principle is rejected more (and the need principle endorsed more) by the Dutch-speakers than by the French-speakers. Why this is the case remains a matter of speculation.

One possibility is that *Groen!* (the Flemish green party) is the most obvious choice for people who want to distinguish themselves from the polarizing Flemish nationalists. For example, contrary to all other mainstream parties, *Groen!* has never joined governments involving N-VA and, in 2014, its voters were the least likely to have voted for N-VA in past elections (Partirep, 2014). Their supporters may therefore favor positions that are more contrasted from those of N-VA than those of their French-speaking counterpart through a selection process. This may also explain the absence of linguistic differences on the Work equity principle (that coheres with the right-wing ideology of the N-VA), in the Green group. By contrast, the French-speaking ECOLO party which has the most volatile electoral base on the French-speaking side (Partirep, 2014), may therefore include a more diverse arrays of political views than *Groen!*

## Departing from Stereotypes

More generally, incorporating political preferences in our statistical model leads us to depart from stereotypical views of the two linguistic groups. On the Dutch-speaking side especially, the Waffle model seems to apply mainly to supporters of the Flemish nationalist party (the most popular with 32% of the votes at the federal elections of May 2014). Hence, linguistic differences *within* political affiliations do not necessarily follow the predictions of the model.

Another reason to question stereotypes lies in the observation that adhesion to these principles is a function of contextual factors. Depending on the intensity of the conflict (*i.e.*, driven by the necessity to form a government), preferences display considerable variations. However, like in a previous study relying partially on the same dataset (Rimé et al., 2015), we find that temporal differences (as a function of generations for Rimé et al. and as a function of year of data collection in the present case), are observed mainly in the Dutch-speaking sample. On the first dimension especially, French-speakers remain remarkably stable across the two measurement times. It is tempting to confront these findings with the historical observation that reforms of the Belgian State have been mainly driven by Flemish demands. Rimé et al. explain their generational effect by the lower salience of a collective memory of Victimization among younger than older Flemings (an explanation that does not apply to French-speakers). Drawing on this interpretation, political conflicts may be more likely to activate memories of past victimization and therefore lead to a greater endorsement of the very justice principles that are associated with these memories among Dutch-speakers.

## Limitations

Our study suffers from several limitations. One concerns the representativeness of the samples, *e.g.*, in terms of age, education and political preferences. Note especially that the number of participants who took part in the two waves of the survey was extremely limited, leaving the possibility that these especially “loyal” participants are not representative of the overall sample. Another useful step would implicate examining more specifically the case of participants from Brussels. Are we justified in grouping Brussels participants with their linguistic groups or is their distinct, regional, approach to this conflict in the capital? Given the small sample size, we did not address this issue in this paper but this question would certainly be worthy of further study.

Moreover, the questionnaire in which this study was embedded was not specifically aimed at testing the Waffle model. A consequence of this state of affairs is that each principle was assessed with only one item. It is clear that a more extensive study should be conducted to properly validate scales evaluating each principle and the relations between these principles.

Also, we did not assess endorsement of equality in the present study (see Klein & Azzi, 2001, for an examination of this principle in the Belgian context). This was purposeful in that equality may have very different meanings when you consider the group or the individual level of analysis. In majority/minority relations, apportioning the same amount of resources to both groups involves breaching equality at an individual level. Conversely, equality at an individual level may be viewed as implying equity at a group level: To the extent that each individual is viewed as an equal contributor to the state, more resources should be apportioned to the majority. This short discussion highlights that the legitimacy of justice principles depends on the level of analysis considered (group vs. individual) and that judgments at these two levels may interact in complex ways.

While we have articulated our interpretation of the present results on the opposition between the two linguistic groups, it is important to address two caveats to this interpretation. First, differences between the two samples may be confounded with socio-economic differences that partially account for the present results. Such a purely economic interpretation should however take into account that the differences we observe are mainly driven by supporters of the NV-A, a party that emphasizes Flemish cultural identity and has little traction on the French side. There is no equivalent of this party on the French speaking side, suggesting that, if economic differences between the two samples impact on justice preferences (e.g., with the more affluent preferring equity), this is not due to sampling (e.g., an over-representation of right wingers in our Flemish sample). Rather, such factors seem to intersect with social and cultural factors that operate at the population level.

Second, it is important to consider that we have assessed adhesion to these principles in the context of relations between the two linguistic groups. Members of the two groups may not exhibit similar differences with respect to the distribution of resources *within their groups*. While the more left-wing orientation of the French speaking part may lead to more intragroup solidarity, greater regional identification in Flanders may exert a similar effect (cf. Hogg & Hains, 1996).

Finally, while we attribute the observation of a greater convergence between 2011 and 2014 to the waning of the conflict surrounding the formation of the government, this interpretation would benefit from direct evidence e.g., through an experimental manipulation of the salience of the intergroup conflict. It is indeed possible that group members’ adhesion to justice principles, while remaining different across group boundaries, de-polarize in a way suggesting that they may occupy center stage in intergroup relations only in conflict escalating periods. This implies that adherence to justice principles, instead of simply being predictors of conflict, may be one of its multiple symptoms or manifestations.

## Conclusions

In conclusion, the present study finds preliminary evidence in support of the Waffle model by showing disagreements between the two linguistic groups along its two dimensions (economic and territorial). It also shows that positions on these two dimensions predict aspirations regarding the future of the country. However, incorporating time and political affiliation shows that the two groups’ attitudes towards these principles are far from stable and homogenous. Dutch and French speakers’ sense of what is fair fluctuates with the ebb and flow of their dissensions.

## Additional File

The additional file for this article can be found as follows:

10.5334/pb.345.s1 Appendix 1 Percentages of Answers for Each Political Party in Belgium in 2011.

## Footnotes

Equality was not evaluated in this study. We explain why and consider this principle in the discussion.

Source: http://economie.fgov.be/fr/statistiques/chiffres/travailvie/emploi/trimestriels/.

The attrition rate was very high between the two waves. The length of the questionnaire, the absence of compensation and our failure to keep in contact with participants between 2011 and 2014 may explain this. In order to examine whether the composition of the sample differed between wave 1 and wave 2, we conducted a binary logistic regression on the 2011 data with the participation in the next wave as an outcome variable and Gender, Language, Political Orientation and Age as predictors. This analysis only revealed an effect of Gender: Men were less likely to drop out than women (84% vs. 89%). Hence, gender was controlled in the analyses.

Changes in degrees of freedom are due to missing values.

Full elections results for the 2010 elections can be consulted here: http://elections2010.belgium.be/fr/cha/results/results_graph_CKR00000.html. A breakup of the sample by political party preference is available in Annex 1 of the Supplementary material.

In terms of provinces: 76 participants reported living in Brussels (16 Dutch-speakers), 31 in Walloon Brabant, 29 in Flemish Brabant (8 French-speakers), 57 in Antwerp (1 French-speaker), 64 in Limburg (4 French-speakers), 16 in Liege (1 Dutch-speaker), 13 in Namur (1 Dutch-speaker), 16 in Hainaut, 5 in Luxembourg (1 Dutch-speaker), 24 in West Flanders and 34 in East Flanders.

FDF is a french speaking party that supports linguistic rights, Vlaams Velang is a far right Flemish party and PTV-PdvA is a far-left, communist, party.
