# Supplementary material for: Principled Disagreements: Adhesion to Intergroup Justice Standards in the Context of the Belgian Linguistic Conflict
Source: Psychol Belg. 2017 Nov 21;57(3):13–31. doi: 10.5334/pb.345 (PMC6194542; doi:10.5334/pb.345)
Supplement: Appendix 1 — Percentages of Answers for Each Political Party in Belgium in 2011. [file pb-57-3-345-s1.pdf]

Appendix 1

Percentages of Answers for Each Political Party in Belgium in 2011

Parties	Percentages	
	French-Speaking	Dutch-Speaking
Dutch-speaking parties		
Groen! (Ecologist)	8.63	16.18
SP.a (Social-Dem)	2.75	10.36
CD&V (Christian- Dem)	1.96	9.06
Open Vld (Liberal)	2.75	9.71
N-VA (Fl. Nationalist)	1.57	38.51
LDD (Populist)	0	1.29
Vlaams Belang (Far right)	0	1.62
French - speaking parties		
Ecolo	24.31	4.85
PS (Socialist)	9.02	1.29
CdH (Center, former Christian-Dem)	12.94	.65
MR (Liberal)	21.18	.65
FDF ("Defense" of French speakers)	8.24	.00
FN (Far right)	.78	0
Other	5.88	5.83
Total Number of Choices	255	309
Total Number of Participants	155	223

Note: as participants could choose multiple political parties, the denominator for these percentages is the total number of choices within each linguistic group, not the total number of individuals.
